# Ventriculoperitoneal Shunt Treatment Increases 7 Alpha Hy-Droxy-3-Oxo-4-Cholestenoic Acid and 24-Hydroxycholesterol Concentrations in Idiopathic Normal Pressure Hydrocephalus

**DOI:** 10.3390/brainsci12111450

**Published:** 2022-10-27

**Authors:** Emanuele Porru, Erik Edström, Lisa Arvidsson, Adrian Elmi-Terander, Alexander Fletcher-Sandersjöö, Anita Lövgren Sandblom, Magnus Hansson, Frida Duell, Ingemar Björkhem

**Affiliations:** 1 Division of Clinical Chemistry, Department of Laboratory Medicine, Karolinska University Hospital, 141 57 Huddinge, Sweden; 2 Department of Neurosurgery, Karolinska University Hospital, Department of Clinical Neuroscience, Karolinska Institutet, 171 77 Solna, Sweden; 3 Stockholm Spine Centre, Department of Clinical Neuroscience, Karolinska Institutet, 171 77 Solna, Sweden

**Keywords:** oxysterols, 27-hydroxycholesterol, 7 alpha hydroxy-3-oxo-4-cholestenoic acid, 24S-hydroxycholesterol, CSF-drainage, idiopathic normal pressure hydrocephalus, ventriculoperitoneal shunt, shunt-treatment, biomarker, cerebrospinal fluid

## Abstract

Idiopathic normal pressure hydrocephalus (iNPH) is the most common form of hydrocephalus in the adult population, and is often treated with cerebrospinal fluid (CSF) drainage using a ventriculoperitoneal (VP) shunt. Symptoms of iNPH include gait impairment, cognitive decline, and urinary incontinence. The pathophysiology behind the symptoms of iNPH is still unknown, and no reliable biomarkers have been established to date. The aim of this study was to investigate the possible use of the oxysterols as biomarkers in this disease. CSF levels of the oxysterols 24S- and 27-hydroxycholesterol, as well as the major metabolite of 27-hydroxycholesterol, 7 alpha hydroxy-3-oxo-4-cholestenoic acid (7HOCA), were measured in iNPH-patients before and after treatment with a VP-shunt. Corresponding measurements were also performed in healthy controls. VP-shunt treatment significantly increased the levels of 7HOCA and 24S-hydroxycholesterol in CSF (*p* = 0.014 and *p* = 0.037, respectively). The results are discussed in relation to the beneficial effects of VP-shunt treatment. Furthermore, the possibility that CSF drainage may reduce an inhibitory effect of transiently increased pressure on the metabolic capacity of neuronal cells in the brain is discussed. This capacity includes the elimination of cholesterol by the 24S-hydroxylase mechanisms.

## 1. Introduction

Idiopathic normal pressure hydrocephalus (iNPH) is a treatable neurological disorder affecting the elderly. The condition is characterized by gait and balance impairments, urinary incontinence and cognitive decline. Diagnostic imaging shows signs of disturbed cerebrospinal fluid (CSF) dynamics, including ventricular enlargement and widened sulci. These findings and the clinical symptoms are thought to reflect impaired CSF outflow and increased intracerebral pressure pulsatility [1,2]. The mechanisms involved in the transport of interstitial fluid and CSF across the blood–brain barrier have not been defined with certainty.

Similarly, the pathophysiology resulting in the triad of iNPH symptoms is also not clearly understood. However, clinical features of the disease include psychomotor slowing, executive dysfunction, impaired attention, decreased verbal fluency and worsened working memory, all of which correlate with the typical patterns of frontal lobe dysfunction [3,4,5]. Radiology studies have demonstrated that patients with iNPH mainly present with a hypoperfusion in the frontal lobe and reduced blood flow in the periventricular area [6,7,8]. Compared to those with Alzheimer’s disease, who also have frontal lobe deficits, iNPH patients generally have milder symptoms.

The symptoms of this disease are potentially reversible if recognized early and treated with a ventriculoperitoneal (VP)-shunt. However, it is notable that the positive effects of the drainage of CSF are not accompanied by a reduction in ventricular size [9].

To evaluate the effect of drainage before VP-shunt surgery, CSF drainage through a lumbar puncture is routinely performed as a diagnostic tool. The patients are evaluated for relevant symptoms including gait, balance, cognition and incontinence before and after the lumbar puncture. The purpose is to identify those that are improved by CSF drainage and consequently have a greater chance of benefitting from VP-shunt treatment. A VP-shunt is a silicone tube with one end placed through the brain into the enlarged ventricles and the other end in the abdominal cavity. The rest of the tube is placed subcutaneously, and a valve defines the flow and pressure gradients needed for drainage.

iNPH is regarded as one of the most misdiagnosed diseases worldwide and may occur with varying combinations or degrees of other neurological conditions, including Alzheimer’s disease, Parkinson’s disease and vascular dementia [2]. Diagnosis is based on symptoms, clinical findings and radiological imaging. However, not all patients respond to CSF drainage and there is a need for improved diagnostic tools and markers to predict treatment outcomes. No distinct laboratory biomarker has been defined to date. However, levels of CSF cortisol, cortisone dehydroepiandrosterone (DHEA), 7 alpha hydroxy dehydroepiandrosterone (7DHEA), 7β-OH-DHEA, 7-oxo-DHEA, 16α-OH-DHEA and aldosterone have been shown to differ between iNPH patients and controls [10]. In the brain 27-OH is metabolized into 7HOCA [11]. 7HOCA may also be produced in extracerebral tissue from 7α-hydroxy-4-cholestene-3-one and naturally occurs in human blood [11,12]. An uptake of 7HOCA from the circulation occurs in the liver, where it is transformed into bile acid [13,14].

**Figure 1:**
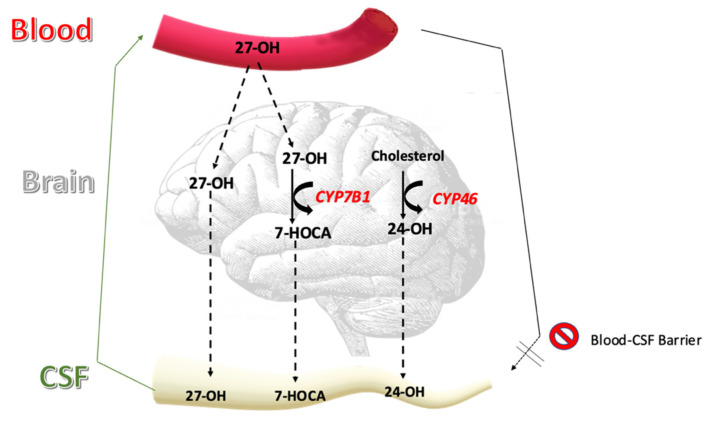
Flux of oxysterols into the cerebrospinal fluid.

The most common oxysterols in CSF include 24S-hydroxycholesterol (24-OH), 27-hydroxycholesterol (27-OH) and the major oxysterol metabolite 7 alpha hydroxy-3-oxo-4-cholestenoic acid (7HOCA) [15,16]. The origin and metabolism of these three sterols are different, and their concentration in CSF is dependent on their sites of primary production, flux across the blood–brain barrier, rate of metabolism in, and elimination from the brain to the CSF [17] (Figure 1).

In humans, 24-OH is almost exclusively formed in the brain and there is a continuous flux of 24-OH from the brain into the circulation. There is also a modest flux of this oxysterol from the brain into the CSF. The 24-OH present in CSF has a mixed origin: part of it originates directly from the brain and part of it originates from the circulation. This means that the integrity of the blood–brain barrier is important for the levels of 24-OH in CSF [18].

There is very little formation of 27-OH in the brain, and most of this oxysterol in the brain and CSF originates from the circulation. Despite the relatively high uptake of this oxysterol by the brain the levels in both brain and CSF are low, reflecting a very efficient metabolism. Given the negative effects of this oxysterol on various brain functions [19,20,21,22,23,24,25,26,27,28], this rapid metabolism can be regarded as a detoxification. The end metabolite of 27-OH in the brain is 7HOCA and there is a net flux of this steroid acid from the brain into the circulation. There is also a flux of this metabolite from the brain into CSF and the concentration in CSF is higher than that of any other oxysterol [15,16]. There is also an extracerebral formation of 7HOCA and there may be a small contribution from the circulation to the content in CSF. Most of the effect of blood–brain barrier defects on the concentration of 7HOCA in CSF is thought to be a consequence of the higher influx of the precursor 27-OH [16]. 

Previous studies have shown normal levels of 7HOCA in patients with Alzheimer’s disease or vascular dementia [16]. In contrast, patients with subdural hematoma or subarachnoid hemorrhage demonstrate an increased level of 7HOCA [14,29,30]. In a review by Sodero, it is suggested that 24-OH has the potential to be used as a stable biomarker for various neurological diseases [31].

Based on the above findings, this study aims to investigate the possible use of the oxysterols 24-OH, 27-OH and 7HOCA as biomarkers in iNPH. In addition, the findings may further our mechanistic understanding of the effects of CSF drainage in iNPH.

## 2. Materials and Methods

### 2.1. Patient Selection and Study Setting

All adult patients (≥18 years) who were offered surgical treatment for iNPH between 2020 and 2022 were eligible for inclusion. The study hospital is a publicly funded and owned tertiary care center, serving a region of roughly 2.3 million inhabitants, and the only neurosurgical center in the region. As part of routine management, referrals to the study center for iNPH were evaluated at a multidisciplinary conference. Cases matching the clinical and radiological criteria for a diagnosis of iNPH were further evaluated in accordance with international guidelines [32], which include scores for gait, balance, incontinence and cognition. At the study hospital, the Hellstrom scale is used for the evaluation of these symptoms [33]. A lumbar tap test was performed to identify patients that improved after CSF-drainage. The tap test is the removal of 30ml of CSF through a lumbar puncture. In addition, a standardized grading of symptoms is performed before and after the removal of CSF. A positive response is defined as an improvement of 5 on a Hellstrom scale [33]. Medical records and imaging data from digital hospital charts were retrospectively reviewed using the health record software TakeCare (CompuGroup Medical Sweden AB, Farsta, Sweden). The study was approved by the National Ethical Review Board (Dnr 2019-04241).

Seventeen patients with iNPH (age 51–88, mean 74) accepted to participate in the study and signed informed consent after having received both written and oral information [32]. CSF samples were collected from 10 iNPH patients before and after treatment with an intraventricular VP-shunt. Seven patients provided CSF only before surgery. Postoperative samples were collected at a median of 4.5 months post-surgery. Twenty-eight patients of similar age, investigated with lumbar puncture for headaches and without pathological CSF findings, were used as controls.

### 2.2. Laboratory Analyses

Albumin was measured using Tina-quant Albumin Gen.2 kit (Roche, Basel, Switzerland). Analytical standards (pure > 98%) of each oxysterol were used for calibration and quantification. 7HOCA was measured by isotope dilution mass spectrometry as described previously [23,30]. The method for 7alpha-hydroxy dehydroepiandrosterone (7DHEA), along with 24-OH and 27OH, was developed and validated to obtain a new comprehensive analytical tool for endogenous oxysterols. The analyses were performed using deuterium labeled 27-OH and deuterium labeled 24- hydroxycholesterol as internal standard. Deuterium labeled 7DHEA was not available as internal standard. Instead, based on our results in terms of precision and accuracy, 27-OH-d5 was used as an internal standard for 7DHEA quantification. In brief, 500 mL of each sample was hydrolyzed by sodium hydroxide solution and heated at 60 °C for 1 h. Following two extractions with 3mL of chloroform, the samples were chemically derivatized by trimethylsylilation prior to analysis by ID-GCMS. Three µL of each sample were injected into a GC-MS system (Agilent GC4890, MS 5973, Santa Clara, CA, USA). The column used was a Agilent J&W HP-5ms 5%-phenyl)-methylpolysiloxane, (0.25 mm × 30 m, 0.25 µm), with the following analytical conditions: an injector temperature of 250 °C, splitless mode, gas flow rate 1 mL/min, oven temperature ramped from 180 °C to 250 in 3.5 min, and subsequently ramped to 300 °C in 12.5 min and held for 9 min, for a total run time of 25 min. The method was validated to satisfy analytical requirement in terms of accuracy (bias < 5%), reproducibility (CV < 5%) and recovery during the extraction step (>95%).

### 2.3. Statistical Analysis

The Shapiro–Wilk test was used to evaluate the normality of the data. As all continuous data significantly deviated from a normal distribution pattern (Shapiro–Wilk test *p* value < 0.05), it is presented as a median (range) and categorical data as numbers (proportion). The Mann–Whitney *U* test was used to compare pre- and postoperative metabolites in the treatment group to non-treated controls (non-matched continuous data), and the Wilcoxon signed-ranks test was used to compare metabolites in each treated patient before and after surgery (matched continuous data). All analyses were conducted using the statistical software program R. Statistical significance was set at *p* < 0.05.

### 2.4. Ethical Aspects

The study was approved by Swedish Ethical Authority and informed consent were obtained for each patient (Dnr 2019-04241). The CSF from anonymous control subjects was obtained from the clinical chemical routine lab. Only information about age and sex was given to the lab analyzing oxysterols.

## 3. Results

The oxysterols 7HOCA, 24-OH, and 27-OH as well as cholesterol, and albumin were measured in 17 iNPH patients and 28 controls. In 10 patients, a repeated analysis was performed at median 4.5 months after VP-shunt surgery.

**Figure 2:**
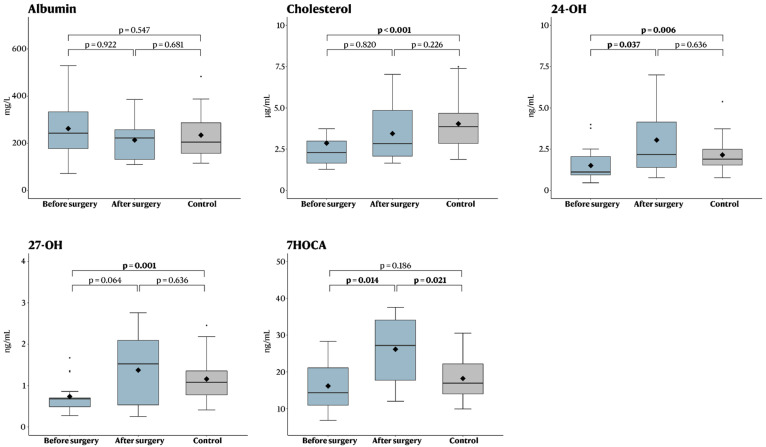
Boxplot showing concentrations before and after surgery, as well as compared to healthy controls. The Mann–Whitney *U* test was used to compare pre- and postoperative metabolites in the treatment group to non-treated controls (non-matched continuous data), and the Wilcoxon signed-ranks test was used to compare metabolites in each treated patient before and after surgery (matched continuous data).

The pre-operative concentrations of cholesterol, 24-OH and 27-OH were significantly lower in iNPH patients compared to controls, while no difference was seen for albumin or 7HOCA (Table 1, Figure 2). For the iNPH patients, surgery was then associated with a significant increase in 24-OH and 7HOCA, as well as a trend towards a significant increase for 27-OH (Table 2, Figure 2). As a result of this increase, post-operative analysis revealed that there was no longer any significant difference in 24-OH and 27-OH between iNPH patients and the control group (Table 3, Figure 2).

## 4. Discussion

In this study, we demonstrate that the CSF levels of 24-OH and 27-OH are lower in iNPH patients compared to controls, and that the levels of the metabolite 7-HOCA significantly increases in iNPH patients after VP-shunt treatment.

It should be emphasized that the major fluxes in oxysterols to and from the brain do not directly include the cerebrospinal fluid, but rather occur between the brain and the circulation. It is believed, however, that changes in these major fluxes as well as metabolic changes in the brain are reflected in the levels of oxysterols in the cerebrospinal fluid [34,35]. 

We hypothesized that measurements of oxysterols in CSF may give useful diagnostic information in iNPH, and that the beneficial effects of CSF drainage may be reflected in changes in the levels of specific oxysterols. Before treatment, the CSF levels of 24-OH and 27-OH were significantly higher in controls compared to iNPH patients (Table 1). VP-shunt treatment affected the fluxes by significantly increasing 7-HOCA. The similar effect of the drainage on all the different oxysterols studied suggests that it is a common factor affecting the fluxes of the different oxysterols. A possible factor is the metabolic capacity of the neuronal cells in the brain [36]. The metabolism of 24-OH by CYP46 and 7-HOCA by the CYP7B1 are dependent on neuronal cells. Since the shunt-treatment led to an increased level of 7HOCA, we would expect reduced metabolism levels of 27-OH. In this study, we did not see a reduction, but rather non-significant changes in the levels of 27-OH.

An increase in the levels of 7HOCA has previously been suggested as a marker for blood–brain barrier dysfunction [16]. In this study, the elevated postoperative 7HOCA levels might be due to the mechanical disruption of the parenchyma during surgery. Nagata et al. demonstrated an increased level of 7HOCA after craniotomy in a patient with subarachnoid hemorrhage [29]. The levels decreased during the first 24 hours post-surgery. However, a significant effect of the surgical procedure may not be so likely in our study, since the levels of 7HOCA were measured several months after surgery. Future studies may benefit from performing serial 7HOCA-sampling in these patients to further understand the mechanisms behind our results.

The levels of 27-OH in CSF are dependent upon the flux in this oxysterol from the circulation into the CSF. Since 27-OH is a precursor to 7HOCA, this is also the case for 7HOCA. The flux of 27-OH across the blood–brain barrier is, however, also combined with a corresponding flux in albumin across this barrier. Albumin is a strong binder of 7HOCA and thus a disrupted blood–brain barrier would be expected to result in increased levels of 7HOCA in the CSF by these two mechanisms [30].

Zhang et al studied the metabolism of oxysterols in cultured rat astrocytes, Schwann cells and neurons. They found that 27-OH, but not 24-OH, was metabolized into 7HOCA in all cell types. Adding 27-OH to the cell cultures resulted in elevated levels of 7-HOCA in astrocytes compered to neurons. Adding 24-OH to the cultures showed an increased metabolization to other cholesterols in astrocytes. This may be of relevance in the context of iNPH, since its pathophysiology may include disturbances in the glymphatic system that particularly affect astrocytes [37,38,39]. If this is the case, astrocytes may recover after CSF drainage and increase the levels of 7HOCA.

Liu et al demonstrated that hypercholesterolemia in the CNS is involved in inducing hydrocephalus [40]. Further research has to be done concerning this, but if the metabolization of 24-OH is affected by hydrocephalus, the increase in 24-OH may occur after VP-shunt treatment due to the increased level of cholesterol accumulated during the development of hydrocephalus.

The increased level of 7HOCA in CSF after VP- shunt treatment is noteworthy, and considering the neurotoxic effects of 27-OH on both neurons and astrocytes [19,28], it seems likely that this effect is beneficial. Further work is needed to evaluate the value of measuring 7HOCA in connection with iNPH treatment.

## 5. Limitations

The main limitation of this study is the limited sample size. Another limitation is the lack of simultaneous analysis of oxysterols in the blood, which could have provided valuable comparative data. This issue needs to be addressed in future studies. A strength of this study is the prospective patient inclusion, as well as the robust collection and analysis of the oxysterol concentrations.

## 6. Conclusions

Neither the etiology of iNPH nor the mechanisms explaining the beneficial effects of VP-shunt surgery are fully understood. This study analyzed CSF-levels of oxysterols before and after VP-shunt surgery, and showed that CSF from iNPH patients compared to controls had a significantly lower level of oxysterols before surgery and that there were significant increases in 24OH and 7HOCA after VP-shunt treatment. CSF drainage may reduce an inhibitory effect of transiently increased pressure on the metabolic capacity of neuronal cells in the brain. This capacity includes the elimination of cholesterol by the 24S-hydroxylase mechanisms. Thus, oxysterols may potentially be used as a biomarker in the diagnosis and management of iNPH.

## Figures and Tables

**Table 1:** Pre-operative metabolites in the treatment group vs. non-treated controls.

Oxysterol	Treatment (*n* = 17)	Control (*n* = 28)	*p*-Value
Albumin (mg/L)	243 (72–529)	205 (115–483), (5 missing)	0.547
Cholesterol (µg/mL)	2.3 (1.3–12), (1 missing)	3.9 (1.9–7.5)	**<0.001**
24-OH (ng/mL)	1.1 (0.5–4.0)	1.9 (0.8–5.4)	**0.006**
27-OH (ng/mL)	0.7 (0.3–1.7)	1.1 (0.4–2.5)	**0.001**
7HOCA (ng/mL)	14 (6.8–28)	17 (10–31), (2 missing)	0.186

Data is presented as median (range). Data compared using the Mann–Whitney *U* test (non-matched continuous data). Bold text in the *p*-value column indicates a statistically significant association (*p* < 0.05). Abbreviations: 24-OH = 24S-hydroxycholesterol; 27-OH = 27-hydroxycholesterol; 7HOCA = 7 alpha hydroxy-3-oxo-4-cholestenoic acid.

**Table 2:** Matched comparisons between metabolites before and after surgery.

Oxysterol	Before Surgery (*n* = 10)	After Surgery (*n* = 10)	*p*-Value
Albumin (mg/L)	215 (72–518)	222 (110–386)	0.922
Cholesterol (µg/mL)	2.5 (1.3–12) (1 missing)	2.8 (1.7–7.0) (1 missing)	0.820
24-OH (ng/mL)	1.6 (0.6–4.0)	2.2 (0.8–7.0)	**0.037**
27-OH (ng/mL)	0.7 (0.4–1.7)	1.5 (0.2–2.8)	0.064
7HOCA (ng/mL)	17 (6.8–28)	27 (12–38)	**0.014**

Data is presented as median (range). Data compared using the Wilcoxon signed-ranks test (matched continuous data). Bold text in the *p*-value column indicates a statistically significant association (*p* < 0.05). Abbreviations: 24-OH = 24S-hydroxycholesterol; 27-OH = 27-hydroxycholesterol; 7HOCA = 7 alpha hydroxy-3-oxo-4-cholestenoic acid.

**Table 3:** Post-operative metabolites in the treatment group vs. non-treated controls.

Oxysterol	Treatment (*n* = 17)	Control (*n* = 28)	*p*-Value
Albumin (mg/L)	222 (110–386) (7 missing)	205 (115–483) (5 missing)	0.681
Cholesterol (µg/mL)	2.8 (1.7–7.0) (8 missing)	3.9 (1.9–7.5)	0.226
24-OH (ng/mL)	2.2 (0.8–7.0) (7 missing)	1.9 (0.8–5.4)	0.636
27-OH (ng/mL)	1.5 (0.2–2.8) (7 missing)	1.1 (0.4–2.5)	0.636
7HOCA (ng/mL)	27 (12–38) (7 missing)	17 (10–31) (2 missing)	**0.021**

Data is presented as median (range). Data compared using the Mann–Whitney U test (non-matched continuous data). Bold text in the *p*-value column indicates a statistically significant association (*p* < 0.05). Abbreviations: 24-OH = 24S-hydroxycholesterol; 27-OH = 27-hydroxycholesterol; 7HOCA = 7 alpha hydroxy-3-oxo-4-cholestenoic acid.

## Data Availability

Data is available from the corresponding author upon reasonable request.

## Abbreviations

24-OH	24S-hydroxycholesterol
27-OH	27-hydroxycholesterol
7HOCA	7 alpha hydroxy-3-oxo-4-cholestenoic acid
DHEA	Cortisone dehydroepiandrosterone
7DHEA	7 alpha hydroxy dehydroepiandrosterone
CSF	Cerebrospinal fluid
CYP7B1	Cytochrome P450 family 7 subfamily B member 1
CYP46	Cytochrome P450 family 46
iNPH	Idiopathic normal pressure hydrocephalus
VP	ventriculoperitoneal
